# Alternating hemiplegia of childhood: a distinct clinical entity and *ATP1A3*-related disorders: A narrative review

**DOI:** 10.1097/MD.0000000000029413

**Published:** 2022-08-05

**Authors:** Piero Pavone, Xena Giada Pappalardo, Martino Ruggieri, Raffaele Falsaperla, Enrico Parano

**Affiliations:** a Pediatric Clinic, Department of Clinical and Experimental Medicine, University Hospital AOU “Policlinico-Vittorio Emanuele”, Catania, Italy; b Unit of Catania, National Council of Research, Institute for Research and Biomedical Innovation (IRIB), Catania, Italy; c Department of Biomedical and Biotechnological Sciences (BIOMETEC), University of Catania, Catania, Italy; d Unit of Rare Diseases of the Nervous System in Childhood, Section of Pediatrics and Child Neuropsychiatry, Department of Catania, Italy, AOU “Policlinico PO San Marco, University of Catania, Catania, Italy; e Unit of Pediatrics, Neonatology and Neonatal Intensive Care, and Pediatric Emergency, AOU “Policlinico”, PO “San Marco”, University of Catania, Catania, Italy.

**Keywords:** alternating hemiplegia of childhood (AHC), *ATP1A3*-related disorders, *ATP1A3* syndrome, *ATP1A3* gene, *ATP1A2* gene

## Abstract

Alternating Hemiplegia of Childhood (AHC) is a rare disorder with onset in the first 18 months of life characterized by stereotyped paroxysmal manifestations of tonic and dystonic attacks, nystagmus with other oculomotor abnormalities, respiratory and autonomic dysfunctions. AHC is often associated with epileptic seizures and developmental delay. Hemiplegic paroxysm is the most remarkable symptom, although AHC includes a large series of clinical manifestations that interfere with the disease course. No cure is available and the treatment involves many specialists and therapies. Flunarizine is the most commonly used drug for reducing the frequency and intensity of paroxysmal events. Mutations in *ATP1A2*, particularly in *ATP1A3*, are the main genes responsible for AHC. Some disorders caused by *ATP1A3* variants have been defined as *ATP1A3*-related disorders, including rapid-onset dystonia-parkinsonism, cerebellar ataxia, pes cavus, optic atrophy, sensorineural hearing loss, early infant epileptic encephalopathy, child rapid-onset ataxia, and relapsing encephalopathy with cerebellar ataxia. Recently, the term *ATP1A3* syndrome has been identified as a fever-induced paroxysmal weakness and encephalopathy, slowly progressive cerebellar ataxia, childhood–onset schizophrenia/autistic spectrum disorder, paroxysmal dyskinesia, cerebral palsy/spastic paraparesis, dystonia, dysmorphism, encephalopathy, MRI abnormalities without hemiplegia, and congenital hydrocephalus. Herewith, we discussed about historical annotations of AHC, symptoms, signs and associated morbidities, diagnosis and differential diagnosis, treatment, prognosis, and genetics. We also reported on the *ATP1A3*-related disorders and *ATP1A3* syndrome, as 2 recently established and expanded genetic clinical entities.

## 1. Introduction

Alternating hemiplegia of childhood (AHC) is an uncommon neurological disorder mainly characterized by paroxysmal transient events of paresis involving either or both sides of the body, usually before the age of 18 months.^[1,2]^ Events are often preceded by precipitating factors such as environmental stress, bathing, and psychological factors. Paroxysmal episodes may occur independently or in association with other clinical manifestations, such as autonomic dysfunction, altered awareness, and abnormal movements, such as dystonia, ataxia, and choreoathetosis. Affected individuals may show developmental delay/intellectual disability (DD/ID) and epileptic seizures.^[1–4]^ Some symptoms tend to occur in sequential distinctive phases and disappear with sleep.^[1–4]^ The prevalence of AHC is 1/1,000,000 in children under the age of 16 years, but this number could be underestimated due to the variability in clinical presentation and the lack of genetic analysis in the preceding epidemiologic data.^[5,6]^ The underlying pathophysiological mechanisms of AHC and/or the various complex co-morbidities are not completely known. Advanced molecular research has allowed a better understanding of causal genes involved and provides, at the same time, early confirmation of the diagnosis. Mutations in *ATP1A2* (AHC1; OMIM#104290) and *ATP1A3* (AHC2; OMIM#614820), which encode two different alpha subunits of the neuronal NA+-K+ ATPase transmembrane ion pump, are the most frequent involved genes.^[7–10]^ AHC individuals with *ATP1A*3 mutations are numerically more common than those with *ATP1A2* mutations.^[11]^ The wider use of genetic technology has enabled it to differentiate AHC diagnosis from other similar disorders and to extend its clinical spectrum. The aim of this study was not only to review the most recent clinical features and genetic data of the AHC disorder with its distinctive clinical and comorbidity variability but especially to report on other conditions linked to *ATP1A3* mutations: *ATP1A3*-related disorders and *ATP1A3* syndrome.

## 2. Methods

This narrative review was performed by collecting clinical trials, primary research, and reviews from online bibliographic databases (MEDLINE, Embase, PubMed, Cochrane Central, Web of Sciences, and Scopus) selected from 2000 to November 2021. The key search terms derived from the medical subject heading terms were pertaining to “Alternating Hemiplegia of Childhood”, “Alternating Hemiplegia of Childhood 1 (AHC1)”, “Alternating Hemiplegia of Childhood 2 (AHC2)”, “*ATP1A3*”, “*ATP1A2*”, “ATP1A3-related disorders”, “*ATP1A3* syndrome”. Relevant studies were manually examined and included in the present reference list. After removing duplicate records, the main search results were included.

## 3. Alternating Hemiplegia of Childhood

### 3.1. Historical annotations

The earliest descriptions of AHC overlapped with some clinical cases with complicated migraine in childhood, resulting in no clear identification. Verret and Steele were recognized as the first authors to describe AHC.^[12]^ They reported 8 patients with AHC and complicated migraine that started in infancy. As the clinical features of AHC became clearer, only 3 of the 8 patients exhibited the classic signs and symptoms of AHC.^[12]^ Later, Dittrich et al.^[13]^ set out some remarkable clinical AHC manifestations such as ocular and dystonic features. Subsequently, Aicardi et al.,^[14]^ Silver and Andermann,^[15]^ Fusco and Vigevano^[16]^ provided a clear and extensive description of symptoms and signs establishing AHC as a clinical entity characterized by a series of attributable symptoms/signs. Aicardi et al.^[14]^ summarized the results of 75 AHC subjects previously described in the literature, indicating clinical features and frequency: hemiplegia involving either side 75/75 (100%); episodes of double hemiplegia/quadriplegia 43/46 (93%); tonic/dystonic attacks 50/52 (96%); oculomotor abnormalities (i.e., nystagmus/gaze deviation/strabismus) 48/49 (97%); disappearance of symptoms with sleep 36/38 (94%); DD/ID 63/64 (98%); and neurological deficits/choreoathetosis/dystonia 49/54 (90%). The same article also showed the diagnostic criteria referred^[14]^ to as: (1) onset before 18 months of age; (2) repeated bouts of hemiplegia involving either side of the body at least in some attacks; (3) other paroxysmal disturbances including tonic/dystonic attacks, nystagmus, strabismus, dyspnea, and other autonomic phenomena occurring during hemiplegic bouts or in isolation; (4) episodes of bilateral hemiplegia or quadriplegia starting either as a generalization of a hemiplegic episode or bilateral from the start; (5) immediate disappearance of all symptoms on going to sleep, with recurrence 10 to 20 minutes after awakening in long-lasting bouts; (4) evidence of DD and neurologic abnormalities including choreoathetosis, dystonia, or ataxia. Further observations in 23 children were indicated by Sakuragawa^[3]^ in Japan. The diagnostic criteria for AHC previously described by Aicardi et al.^[14]^ were slightly adjusted by Mikati et al.^[1]^ as follows: episodes of hemiplegia alternated between one side and the other; episodes of movement disorders, such as dystonia, choreoathetosis, ataxia, and intermittent abnormalities in eye movements; and evidence for an encephalopathic process, including developmental and cognitive delay, with or without spastic diplegia, spastic quadriplegia, or hypotonia. Particularly with regard to the natural history of AHC, Silver and Andermann^[15]^ delineated that hemiplegic attacks were noted at a mean age of 9 months, and the AHC diagnosis was reached at a mean age of 4.8 years. Dystonic attacks and nystagmus were the early symptoms, which tended to reduce in frequency after the first year of life, while after this age, hemiplegic episodes showed an increase in frequency.^[15]^ At the beginning of the 2000s, the progressive use of genetic mutation analysis confirmed the diagnosis of AHC giving a relevant boost to the knowledge of this disorder and its comorbidities.^[6,9,17–19]^

### 3.2. Symptoms and signs

Clinical manifestations vary with the grade of severity, type of complications, and associations with other morbidities. Episodes have an early onset, usually in the first months of life, and are often preceded by trigger events. The most frequent initial clinical manifestations are tonic or dystonic episodes, ocular abnormalities, and autonomic phenomena often linked to trigger events. Paroxysmal tonic/dystonic episodes are unilateral, appearing with the extension of one limb and, more rarely, with one side of the body. Ocular abnormalities mainly consist of nystagmus affecting only one eye, which may occur as an isolated manifestation or in association with tonic or dystonic signs.^[14]^ Autonomic phenomena such as vasomotor changes may also be observed.^[3–5]^ Incorpora et al.^[20]^ described monozygotic twins who complained of hot water reflex seizures until 3 years each time they were immersed in a warm water bath or when hot water was sprinkled over the body eliciting irritability, unprovoked smile, head deviation, and upper-limb hypertonia to one side lasting from a few seconds to 3-4 min. These episodes ceased after crying. Guttural sounds and noisy breathing were observed. At 30 months, after a previous episode of an acute encephalopathy, both twins showed the typical signs of AHC, having numerous spontaneous episodes of dystonic movements prevalently on the right side together with ataxia, eye deviation, and autonomic disturbances. In its classical manifestations,^[1–4]^ the hemiplegic episodes are light and inconsistent at onset, but then gradually assume gradually the typical clinical manifestation. Hemiplegia may start abruptly or progress over several minutes affecting one side and less frequently both sides. Episodes occur with variable frequencies and may last from a few minutes to several days. Consciousness is preserved during an attack, and the child is irritable and fretful. The arms were more severely affected than the lower limbs. During the same episode, hemiplegia may shift from one side to another. A hemiplegic event is followed by marked weakness. In association with hemiplegic episodes, tonic and dystonic movements, choreoathetosis, ataxia, nystagmus, and oculomotor abnormalities are often observed.^[1,2,5–8]^

One of the largest AHC studies was conducted by Sweney et al.^[4]^ who reported 103 individuals (56 females and 47 males). According to these authors,^[4]^ the most frequent and early symptoms observed in the first 3 months of life were paroxysmal eye movements in 83% and hemiplegic episodes by 6 months in 56% of patients. Dystonic symptoms preceded hemiplegic episodes in 35/86 (41%) cases, and both dystonic and plegic episodes had a co-occurrence onset in 30/86 (35%). The duration of dystonic and plegic episodes was extremely variable, ranging from minutes to hours to days. Episodes of abnormal eye movements were reported in 96/103 (96%); nystagmus was the most frequent in 43/96 (50%) and on one side in 10/43 (23%). Intermittent cases of esotropia/exotropia or other monocular deviations were observed in 40/96 (41%). Moreover, neurologic comorbidities including epilepsy were detected in 44/103 (43%), while cognitive involvement (from mild to moderate) was found in 96/96 (100%). The neuropsychological evaluation performed in 41 cases showed a high variability in the functional impairment for the cognitive, adaptive, and behavioral domains, which appeared more severe in older patients. Persistent ataxia was documented in 88/92 (95%) of the cases.^[4]^ The long-term outcome of individuals with AHC was assessed by Bourgeois et al.^[21]^ describing that the paroxysmal nystagmus is more likely to disappear before 10 years, while paroxysmal episodes of hemiplegia tend to persist at a high frequency. In addition to these, tonic, dystonic, and unilateral or bilateral paroxysms often present with high-intensity and painful sensations. In fact, Bourgeois et al.^[21]^ described that one of their patients died of bronchopneumonia after an episode of status epilepticus. Seven of the 9 patients suffered from epilepsy, DD/ID, and severe language impairment. Besides, the gait incoordination persevered and 3 of them also showed signs of persistent hypotonia on the side mostly affected by hemiplegic attacks. They lived a normal life but needed continuous clinical assistance.^[21]^ In another work, Bourgeois evaluated on 29 AHC individuals a progressive reduction in intensity and frequency of the paroxysmal hemiplegic episodes while dystonic movements and choreoathetosis were constantly present.^[22]^ The data collected by Panagiotakaki et al.^[6]^ in a large cohort of 157 patients with a median age of 20 months, ranging from 9 months to 52 years revealed that all patients experienced hemiplegic attacks, among which 86% of them had episodes of bilateral weakness, 88% had dystonic attacks, 53% had epileptic seizures, 72% developed chorea and/or dystonia, and 92% had cognitive impairment. The severity of symptoms was stable, except for abnormal ocular movements and hypotonia, which regressed till disappearance into adulthood. Seven patients died due to severe plegic attacks or episodes of epileptic seizures.^[6]^ The issue of beneficial effects of sleep, as underlined by Aicardi et al.^[14]^ and Bourgeois^[22]^ has been widely demonstrated. As also confirmed by Ricci^[23]^ in a study of 4 children, the sleep structure, duration, cycle length, REM latency, and REM and slow-wave sleep percentages yielded normal results in all children. Besides, as usually reported in AHC individuals, the results of laboratory analysis for metabolic disorders, brain magnetic resonance imaging (MRI), angiographic MRI, CT scan, and liquoral examination are normal. The variability in the clinical expression of paroxysmal and nonparoxysmal episodes in AHC patients is well known.^[24]^ Clinical manifestations associated with hemiplegic events may be correlated to different gene mutations or to the influence of epigenetic factors.^[25]^ It is possible to observe an intrafamilial clinical variability as shown by Pavone et al.^[26]^ following the clinical course and long-term outcomes of AHC in twin sisters. In these twins, clinical manifestations of AHC started in the early days of life with episodes of bath-induced abnormal ocular movements that persisted from the first months to 2 years, together with DD. Preceded by episodes of acute encephalopathies, the twins at 2–3 years had paroxysmal hemiplegic events until 11 years when the hemiplegic attacks tended to regress in association with a gradual increase of migraine episodes. The twins did not have a similar clinical course with regard to the intensity and frequency of hemiplegic episodes, migraine attacks, and epileptic seizures, which were more pronounced in one of the twins. The cognitive impairment was mild in both twins. Recent diagnostic criteria were dictated by Rosewich et al.^[18]^ and Mikaki et al.^[24]^ who found that most AHC individuals show a variable degree of associated disorders such as neuropsychological abnormalities, DD/ID, epileptic seizures, motor dysfunction, abnormal movements, migraine, sleep, and cardiac disturbances.^[5–8]^

### 3.3. Clinical manifestations besides the hemiplegic events

#### 3.3.1. Neuropsychological disturbances.

Jasien et al^[27]^ evaluated neuropsychological abnormalities in 25 AHC individuals and found significant impairments in cognition, expressive and receptive language, executive function, attention, and behavior. In 20 of these patients, 10 showed attention-deficit/hyperactivity disorder (ADHD), 7 had disruptive behavior, and 3 had an anxiety disorder. Eight out of 25 subjects exhibited slower difficulty in comprehension, poor memory, inadequate academic performance, difficulty in math, and difficulty with self-help skills. Concerning the intellectual functioning was normal in 4/25 (16%), borderline in 3 (12%), and impaired in 18 (72%), of which in 6 (24%) were mild, in 10 (40%) moderate, and in 2 (8%) severe.^[27]^

#### 3.3.2. Cognitive impairment.

In 22 individuals observed by Aicardi et al.^[14]^ 5 had midly-delayed or borderline intelligence, 13 had moderate to severe cognitive difficulties, and 4 patients were not examined. In a report by Sakuragawa,^[3]^ cognitive impairment was found in 20 of 23 (87%) patients. Cognitive impairment was also observed by Mikaki et al.^[1]^ in 40 out of 44 (90%) patients suggesting that the cognitive dysfunction correlated with the age of patients and with the age of onset of the hemiplegic attacks. This raised the doubt whether the cognitive impairment in AHC individuals is a primary effect of the disorder or linked to the paroxysmal episodes. Cognitive deficits of various degrees were found in 29 patients reported by Bourgeois^[22]^: in 8 children was mild or borderline level, and in 21 moderate to severe. According to this study,^[22]^ the cognitive impairment was slow during the first 2 years of life and became more marked subsequently to finally reach a plateau before age 10. Polanowska et al.^[28]^ performed neurological examinations in 2 adult patients and found normal or near-normal cognitive functioning with only isolated executive dysfunctions.

#### 3.3.3. Epilepsy.

Epileptic seizures are rarely observed at the onset of AHC signs, but during the course of the disorder, they are reported in about 50% of the cases.^[6,15]^ In the study by Silver and Andermann,^[15]^ epileptic events were reported in 5 out of 10 (50%) patients, one of whom suffered from convulsive status epilepticus, and by Saguragawa^[3]^ in 6 out of 9 (66%) AHC patients. Among 44 out of 103 (43%) AHC patients recorded by Sweney et al.,^[4]^ the seizures were generalized, tonic, or tonic-clonic types, and the mean age of epileptic onset attacks was reported to be around the age of 6 years. Ten children (23%) did not show further epileptic episodes until the age of 10 years or older. Epileptic seizures of generalized, tonic, or clonic types were reported by Saito et al.^[29]^ in 7 out of 9 (77%) AHC patients aged between 2 and 16 years, while ictal epileptiform discharges were detected only in 4 patients which complained of status epilepticus, repeated several times in 3 of them. Thirty-two of the 51 (62%) AHC patients described by Uchitel et al.^[30]^ had epilepsy; in 18, the seizures were focal and mainly frontal. In 11 patients, seizures were generalized specifically, tonic-clonic, myoclonic, and/or absent. In 8 of these cases, the seizures preceded the other paroxysmal events. Epileptic seizures were also detected in 8/44 (19%) by Mikati et al.,[1] in 4/9 (44%) by Rosewich et al.,^[18]^ and in 2/4 (50%) by Pavone et al.^[31]^

#### 3.3.4. Motor dysfunction.

Delays in motor milestones were frequently observed. Bourgeois^[22]^ reported in her study that independent walking in 24 subjects was achieved at an average age of 44 months, as well as poor fine motor control. The gross motor hypotonia was often observed from the onset.^[22]^ Another study conducted by Masoud et al.^[32]^ in 23 AHC patients (9 males, 14 females, mean age 9 years and 4 months) was aimed to analyze gross motor, upper extremity motor control, motor speech, and dysphagia functions. According to their results,^[32]^ motor speech deficits were more severely affected than gross motor abnormalities measured by the Gross Motor Function Classification System (GMFCS). Using GMFCS criteria, 20 patients were in the less than moderate category and only 3 in the moderate to severe category. Another important finding from this study^[32]^ was recognizing that the oropharyngeal function is the most severely affected brain domain in patients with AHC.

#### 3.3.5. Abnormal movements.

Choreoathetotic movements with various levels of intensity as well as ataxia, which were essentially static type, were observed in all cases reported by Aicardi et al.^[14]^ Choreoathetosis was observed in 22 (50%), ataxia in 30 (68%), and dysarthria in 29 (68%) of 44 cases reported by Mikati et al.^[1]^

#### 3.3.6. Migraine.

It is a sign that is not commonly reported in patients with AHC or in family history. Migraine in family history was found in 2 out of 22 (9.1%) and in 3 cases as prodrome of the attacks in the patients reported by Sakuragawa.^[3]^ A family history of migraine in immediate family members was reported by Mikati et al.^[1]^ in 25% of the patients. A single case of migraine out of 10 was reported by Saito et al.^[29]^ in a boy of 15 years old.

#### 3.3.7. Sleep.

The clinical beneficial effect of sleep in this disorder has been widely demonstrated,^[14,22]^ but also the sleep dysfunction has been commonly reported in AHC subjects. In a study by Kansagra et al.,^[33]^ 20 out of 22 AHC patients showed at least one type of sleep problem: 6 complained of obstructive sleep apnea syndrome, an abnormal mean overall apnea-hypopnea index, and an abnormal mean arousal index. Again, 16 had difficulty in falling asleep and staying asleep, or both, while 9 suffered from insomnia and 2 had delayed sleep-wake syndrome.

#### 3.3.8. Cardiac dysfunction.

Cardiac conduction abnormalities in AHCs have also been reported. Nakashima et al.^[34]^ showed large fluctuations in heart rate variability, including low-and high-frequency components in a 20-year-old female while sleeping, but fluctuation rates were suppressed during paralytic attacks.

#### 3.3.9. Other manifestations.

Paroxysmal manifestations of tonic or dystonic attacks, autonomic dysfunction such as dyspneic episodes, vasomotor changes, hyperventilation, hypoventilation/apnea, abnormal eye movements, and monocular-isolated nystagmus may often be observed in AHC patients.^[14,22,29]^

### 3.4. Differential diagnosis

Clinical manifestations in individuals with AHC may overlap with those of other severe or benign disorders. In the differential diagnosis, moyamoya angiopathy and mitochondrial diseases, such as Kearns Sayre syndrome (KSS), mitochondrial encephalomyopathy, lactic acidosis, and stroke-like episodes (MELAS) should be excluded. Moyamoya angiopathy is a vascular disorder characterized by progressive stenosis of the terminal portion of the internal carotid arteries and the development of a network of abnormal collateral vessels for which the carotid artery becomes blocked or narrowed, reducing cerebral blood flow. This anomaly leads to brain ischemic events in children and strokes in adults. The disorder may present with transient ischemic attack and visual disturbances, aphasia, weakness, numbness, or paralysis involving face, arm, or leg typically in one side of the body mimicking the AHC episodes.^[35]^ KSS is a subtype of chronic progressive external ophthalmoplegia (CPEO) manifesting with a triad of onset before the age of 20, CPEO, and pigmentary retinopathy. Individuals affected may also show other clinical features such as complete heart block, high level of protein in cerebrospinal fluid, cerebellar ataxia, deafness, intellectual delay, and endocrine abnormalities.^[36]^ MELAS presents in children or young adults as recurrent episodes of encephalopathy, myopathy, headache, and focal neurologic deficits slowly progressive.^[37]^ Clinical differentiation of AHC should include other disorders of the group of *ATP1A3*-related disorders such as early-onset infantile epileptic encephalopathy, rapid-onset dystonia-parkinsonism, and others.^[38,39]^ Benign familial nocturnal alternating hemiplegia of childhood (BNAHC) is considered a probably migraine-related disorder presenting with recurrent attacks of hemiplegia arising from sleep, and observed in children in the absence of neurological or cognitive impairment.^[40]^ Among the 2 personal cases and 12 collected by the literature, Maas et al.^[41]^ stated that the age of onset of BNAHC ranged from 4 months to 3 years. Episodes of hemiplegia occurred during nocturnal or daytime sleep and were associated with inconsolable crying. Familial or sporadic hemiplegic headache is a subtype of migraine with aura. Individuals with this disorder experience weakness on one side of the body just before or during migraine headaches, and the degree of weakness can vary from mild to severe. Weakness is also associated with other types of aura and visual symptoms. Weakness may affect only a part of the body and, more rarely, the entire body.^[42]^

### 3.5. Treatment

The treatment is complex as it considers all various aspects of AHC. There is no cure for individuals affected by AHC, but treatment with flunarizine may help to control symptoms. AHC treatment may regard acute management of the attacks and prophylaxis of the episodes.^[5]^ A variety of medications have been proposed for the treatment of episodic attacks, but calcium channel blockers have been demonstrated to be the most effective. The most common drug is flunarizine at a dose of 5–20 mg/day, which is more frequently used at 10 mg. Acute treatment consists of removing the known triggers and facilitating early sleep. Silver and Anderman,^[15]^ and Casaer^[43]^ found that flunarizine treatment reduced the duration and severity of hemiplegic attacks, but only infrequently reduced the duration and severity of symptoms. Flunarizine was used in the treatment of AHC in 17 children with AHC by Bourgeois and Aicardi,^[44]^ who reported a significant decrease in the frequency of hemiplegic attacks (by more than 50%) in only one case, while 9 of the children had a significant decrease in the severity and duration of the hemoplegic attacks, with an average duration reduced from several days to a few hours. According to a large Japanese cooperative study of 23 cases of AHC, flunarizine was effective in decreasing the frequency and severity of the events in three-fourths of the cases.^[45]^ In the study by Mikaki et al.^[1]^ 27 out of 44 AHC patients treated with flunarizine, a clinically worthy reduction in the frequency and/or severity was obtained in 21 patients (78%); flunarizine eliminated the hemiplegic attacks completely in 1 patient (4%), partially improved the attacks in 2 patients (7%), and was not effective in four patients. Sweney et al.^[4]^ obtained improvement in dystonic and hemiplegic episodes in 48 of 80 patients using flunarizine, and 21 of 55 patients using benzodiazepines. Several agents such as benzodiazepines, topiramate, ketogenic diet, triheptanoinsteroid, oral adenosine-5’-triphosphate supplementation, amantadine, memantine, aripiprazole, coenzyme q, acetazolamide, dextromethorphan, and vagus nerve stimulator have been used but with not complete effectiveness.^[46]^ Neville and Ninan^[47]^ suggest at the onset of each attack, the use of flunarizine, the treatment of the epileptic seizure, in attempting to avoid trigger situations, and rapid advice to sleep. In a cohort of 30 patients, Pisciotta et al.^[11]^ report the use of flunarizine as the most common treatment, which offers good results reducing duration and frequency of attacks in 50% of patients and decreased intensity in 32%, with major efficacy in younger patients. The efficacy of topiramate was reported in four patients by Jiang et al.^[48]^ The drug significantly improved the frequency and duration of hemiplegic attacks in all patients. A good response was also observed in AHC with additional symptoms, such as seizures, migraine, involuntary movements, autonomic symptoms, and impaired mental development. Van Hillegondsberg and Michaelis^[49]^ with the use of verapamil 6.6 mg/kg/die in 3 divided doses obtained an effective response in a 5-year-old boy with regard to frequency, severity, and duration, and after 6 months of free major debilitating episodes. Samanta and Ramakrishnaiah^[50]^ treated 2 patients with AHC with intravenous immunoglobulin (IVIG) infusion. An 8-year-old girl was treated for 4 years with periodic IVIG infusion, which resulted in free paroxysmal events during the first 16 months of treatment and a boy 2-year-old receiving IVIG infusion for 10 months remained seizure-free for 2 years since the beginning of treatment, but poor success was achieved with hemiplegic episodes.

### 3.6. Prognosis

AHC is a complex, heterogeneous disorder in which the hemiplegic episodes are only a sign, even if the more relevant of several other clinical dysfunctions mainly involving the autonomic nervous system, musculoskeletal system, and brain with seizures and cognitive impairment. AHC patients may show various symptoms, mild or severe manifestations, and associated disorders. Any of these dysfunctions may have an important long-term effect on children with AHC. The episodes of hemiplegic episodes tend to decrease as the child gets older,^[1,2]^ and in a recent study by Cordani et al.,^[51]^ epileptic seizures were found in 24/39 (62%) of AHC patients, which may have a deleterious role in the course of the disorder. particularly if seizures are drug-resistant. There is no proof that AHC limits life expectancy, but patients may complain of complications, such as aspiration, which may be life-threatening. When present, migraine, fine and gross motor dysfunction, walking disturbances, speech, and cognitive impairment tend to persist.

### 3.7. Genetics of alternating hemiplegia of childhood

Two forms of the disorder, AHC1 and AHC2, depend on mutations in *ATP1A2* and *ATP1A3*, respectively, with over 75% of AHCs caused by a mutation in the *ATP1A3* gene. The Na+/K+-ATPase pump is partly responsible for establishing and maintaining electrochemical gradients of sodium and potassium ions across the plasma membrane of neurons. The α3-isoform is primarily found in the nervous system and is considered the most common form of the α-subunits of the basal ganglia, hippocampus, and cerebellum. Despite the growing number of pathogenic variants described, the most extensive cohort studies conducted in various populations have shown that 3 variants account for approximately 60% of all cases.^[51]^ The p.Asp801Asn variant is detected in 30%–43%, p.Glu815Lys is responsible for 16%–35%, and p.Gly947Arg is responsible for 8%–15% of cases.^[6,52,53]^ Other gene mutations (CACNA1A, SLC1A3, and SLC2A1) have occasionally been related to AHC showing similar clinical presentations.^[37,54–56]^ Basically, these genes belong to the class of membrane carriers, which modulates the regulation of the neurotransmission (*CACNA1A*, *SLC1A3*) and the energy metabolism (*SLC2A1*). Many evidence identified a link bewteen alterations found in the calcium voltage-gated channel subunit alpha 1 (*CACNA1*A; OMIM#601011) and in the glutamate transporter excitatory amino acid transporter 1 (*SLC1A3*; OMIM#600111) with the impaired neurotransmitter release in a wide range of neurodevelopmental disorders, among which are episodic ataxia, type 2 (OMIM#108500); migraine, familial hemiplegic, 1 (OMIM#141500); and spinocerebellar atxia 6 (OMIM#183086).^[54,55]^

Mutations in the gene solute carrier family 2 member 1 (*SLC2A1*; OMIM#138140) encoding the glucose transporter, GLUT-1, are known to cause the Glut1 deficiency syndrome (Glut1DS, OMIM#606777) mainly characterized by intellectual disability, seizures and ataxia, leading to a wide range of phenotypes clinically overlap between Glut1DS and AHC patients.^[56]^

## 4. AHC and *ATP1A3*-related disorders

Mutations in *ATP1A3* are more frequent causes of AHC, presenting in about 78% of the cases^[10]^ and 92% in a recent Italian study.^[51]^ Aside AHC and *ATP1A3* mutations have been linked to other clinical entities, such as *ATP1A3*-related disorders. These disorders represent a clinical continuum that includes various distinct entities: AHC, rapid-onset dystonia-parkinsonism (RPD),^[18]^ and cerebellar ataxia, areflexia, pes cavus, optic atrophy, sensorineural hearing loss (CAPOS) syndromes.^[57]^ Subsequently, the clinical spectrum of *ATP1A3*-related disorders has been further expanded, including early infantile epileptic encephalopathy (EIEE),^[58]^ relapsing encephalopathy with cerebellar ataxia (RECA),^[59]^ and childhood rapid-onset ataxia (CROA).^[60]^ Intermediate phenotypes or affected individuals with atypical features that do not completely express classical signs of the above-mentioned disorders have been reported.^[61–63]^

### 4.1. Rapid-onset dystonia-Parkinsonism

Rapid-onset dystonia-Parkinsonism (RDP) was first described by Dobyns et al.^[64]^ in a large family affected by autosomal dominant dystonia-Parkinsonism. Based on the unusually rapid evolution of signs and symptoms, the authors decided to call the disorder RDP. In this work, the age of onset of the disorders ranged between 14 and 45 years. In 6 affected individuals, the onset was acute, presenting over the course of several hours, and in four with subacute onset with evolution over several days or weeks. The progression of symptoms is usually very slow.^[64]^ The association of RDP with *ATP1A3* as a pathogenic variant was reported by de Carvalho et al.,^[65]^ who described the finding of 6 missense mutations in the gene *ATP1A3* in 7 unrelated families. Clinically, the disorder starts abruptly over hours to several weeks with bulbar dysfunction, more often with dysarthria, hypophonia, and mild to moderate dysphagia. Cranio-cervical and limb dystonia, bradykinesia, and postural instability were not responsive to L-dopa, observed.^[63]^ Motor dysfunction remains fixed over time, but second episodes of abrupt worsening have been reported by Cook et al.^[66]^ in 58 RDP subjects, 29 with *ATP1A3* mutations, and 29 control subjects without mutation. Movement disorder was assessed using the Burke-Fahn-Marsden Dystonia Rating Scale (BFMDRS), Unified Parkinson’s Disease Rating Scale (UPDRS), and a cognitive battery of learning, memory, psychomotor speed, attention, and executive function assessments. Among the RDP patients, the majority had motor symptom onset by the age of 25 years with the initial symptoms in the upper body (face, mouth, or arm). Dystonic-parkinsonism was confirmed by the results of BFMDRS (mean+SD,52.1+/± 29.5) and UPDRS motor subscore (29.8+/−12.7).^[66]^ Symptoms are usually triggered by physical or psychological stressors, including exercise, emotional stress, and overheating. Haq et al.^[67]^ in a cohort of 50 *ATP1A3* mutation-positive individuals (carriers) and 44 mutation-negative family members obtained the following results: symptoms began focally but progressed to be generalized in 51% or multifocal in 49% of the cases, with arm onset (41%) as the most common. The arms and voices were more severely affected. Other symptoms observed in the cohort consisted of dystonia with rapid onset, Parkinsonism, bulbar symptoms, headache, seizures mainly focal frontal, and a history of mood disorder and psychosis. Triggers preceded onset in 77% of the cases.^[67]^ The clinical diagnostic criteria for RDP proposed by Rosewich et al.^[18]^ include: (1) onset of dystonia in Parkinson’s disease over a few minutes to 30 days; (2) a clear rostrocaudal gradient of involvement (face>arm>leg); (3) prominent bulbar findings on examination; (4) absence of response to an adequate trial of L-dopa; (5) family history consistent with autosomal dominant inheritance, but de novo mutations are increasingly well documented.

### 4.2. Cerebellar ataxia, areflexia, pes cavus, optic atrophy, sensorineural hearing loss

Nicolaides et al.^[68]^ reported 3 members of a family who showed clinical features, including relapsing, early-onset cerebellar ataxia, associated with progressive optic atrophy, and sensorineural deafness. The patient also presented with areflexia without signs of peripheral neuropathy and pes cavus deformity with varying degrees of severity. Extensive neurological investigations carried out on these patients resulted in normal.^[68]^ Demos et al.^[57]^ in 2014 identified in a proband affected by cerebellar ataxia, areflexia, pes cavus, optic atrophy, sensorineural hearing loss (CAPOS) syndrome, and in his affected sister and mother a novel heterozygous missense mutation, c.2452G> A (p.Glu818Lys), in the *ATP1A3* gene. According to Sharawat et al.^[69]^ the onset of symptoms typically starts between 1 and 5 years, with a mean age of onset of 2.5 years. Most episodes are triggered by fever and characterized by encephalopathy, hypotonia, paresis, abnormal eye movements, and cerebellar ataxia. Classical manifestations include after the acute episode of sensorineural hearing loss, areflexia, present in 97% of the cases, cerebellar ataxia (94%), optic atrophy (91%), nystagmus (44%), dysarthria (37%), and pes cavus (34%).^[57]^ A relapsing course of ataxia encephalopathy (1–3 episodes) with slow progression has been reported.^[69]^

### 4.3. Early infantile epileptic encephalopathy

EIEE was first described by Ohtahara et al.^[70]^ in 1987, by Yamatogy and Ohtahara^[71]^ in 2002, and subsequently reported in 16 affected children. The epileptic disorder is clinically characterized by early-onset seizures in 30% of cases within the first 10 days of life, presenting with generalized and symmetrical or lateralized tonic spasms with less frequency by focal and myoclonic features. Tonic spasms occur in waking and sleeping states. Intercritical EEG shows high-voltage bursts of slow waves mixed with multifocal spikes intermixed with phases of the suppression-burst pattern.^[72]^

Novel heterozygous mutations (p.Gly358Val and p.Ile363Asn) in *ATP1A3* were reported in 2 children by Panagiotakaki et al.,^[6]^ who showed severe epileptic seizures. In one child, seizures started a few hours after birth with high recurrence, marked resistance to treatment, and frequent evolution to status epilepticus. Postnatal microcephaly was reported, and MRI showed progressive brain atrophy. One child had a shortened survival and exitus occurred at the age of 16 months, and the child suffered from prolonged episodes of apnea that required tracheostomy and ventilator support for many months. The patient developed postnatal microcephaly and severe cognitive impairment. Three children with features resembling those reported by Panagiotakaki et al.^[6]^ were described by Marzin et al.^[73]^ as presenting de novo p.Asp742Tyr, p.Cys346Arg, and p.Asp609Tyr, respectively sequence variants in *ATP1A3.* All 3 children showed early-onset epilepsy, movement disorders unrelated to epilepsy, and severe DD. No hemiplegic attacks were observed in these children. Two girls, one with a p.Gly89fs carrier in *ATP1A3* mutations and the other with a p.Gly706Arg *ATP1A3* variant, were reported by Holze et al.^[74]^: the girls complained since the first year of life of severe episodes of apnea that the authors maintained as early-onset autonomic seizures related to the underlying pathogenetic *ATP1A3* variants.

### 4.4. Childhood rapid onset ataxia

Sweadner et al.^[60]^ described a 21-year-old male who presented with ataxia and dysarthria that appeared over a period of months. The patient’s personal history was uneventful except for mild amblyopia, learning disability, dyslexia, and episodes of vertigo that started at the age of 19 years. On clinical evaluation, the patient showed many characteristics of RDP but with minimal fixed dystonia, and brain MRI showed signs of progressive cerebellar atrophy. Genetic analysis showed an *ATP1A3* p.Gly316Ser mutation. Instead, a 28-year-old male was reported by de Gusmao et al.^[75]^ with a history of mild learning disability and migraine who at the age of 21 years started to develop falls without any specific trigger. The signs progressed to marked gait and appendicular ataxia associated with dysarthria, action tremors, and myoclonus. Oculomotor abnormalities were also observed without optic nerve or retinal pathologies. Within a few days, the patients became walker-dependent and wheelchair-dependent for longer distances. De novo mutations were found in exon 8 (c.946 G>A, p.Gly316Ser). Furtherly, 3 cases of CROA due to 2 different *ATP1A3* variants were observed by Schirinzi et al..^[76]^ Two of three patients were mother and son, both found with the variant c.2266C>T (p.R756C), and the third patient carrying the c.2452G>A (p.E818K) variant, this latter commonly described in patients with CAPOS syndrome. Ataxia is increasingly reported in *ATP1A3*-related disorders, and thus should be considered a peculiar clinical feature of this condition.

### 4.5. Relapsing encephalopathy with cerebellar ataxia

A 34-year-old female patient with a heterozygous *ATP1A3* p.Arg756Cys variant was reported by Dard et al.^[77]^ This patient showed relapsing encephalopathy with recurrent episodes of cerebellar ataxia and altered consciousness during the febrile illness. Hully et al.^[78]^ described 2 unrelated sets of full siblings in 2 unrelated families, both of which displayed parental germline mosaicism. In the first family showing (c.2116G>A) p.Gly706Arg *ATP1A3* mutations, the brother and sister presented with severe intellectual deficiency, early-onset drug-resistant epilepsy, ataxia, and autistic features. In the second family (c.2266C>T) p. Arg756Cys, both sisters showed severe encephalopathy with ataxia and dystonia, followed by regression during a febrile episode. Eight new pediatric cases of RECA caused by variants involving p.Arg756 in *ATP1A3* mutations were characterized by Sabouraud et al.^[59]^

## 5. *ATP1A3* syndrome

### 5.1. *Fever-induced paroxysmal weakness and encephalopathy*

A group of patients with *ATP1A3* R756H mutation was reported by Yano et al.^[79]^ In these patients, the onset started in childhood with infrequent fever-triggered paroxysms of encephalopathy and weakness, mostly generalized with slow improving but persistent deficit. Bulbar and oculomotor impairments were also been reported. The authors^[79]^ also indicated that the long-term outcomes may vary from mild motor apraxia to dysphagia. In addition to this, they sustained that some features such as dysarthria, cognitive impairment, motor apraxia, and gait difficulties may also be observed.

### 5.2. Slowly progressive cerebellar ataxia without paroxysms

Only from Sasaki et al.^[80]^ there are findings of 2 patients carrying 2 different *ATP1A3* variants, c.460A>G(p.Met154Val) and c.1050C>A(p.Asn350Lys), who showed slow progressive cerebellar ataxia in the infantile period in the absence of paroxysms or episodic symptoms. The brain MRI presented mild cerebellar cortical atrophy in both.

### 5.3. Childhood-onset schizophrenia/autistic spectrum disorder

Childhood-onset schizophrenia (COS) is a rare and severe disorder that occurs before the age of 13 years. A 9-year-old boy with a de novo mutation c.385G>A in *ATP1A3*, having a history of selective mutism, severe behavioral disturbances, aggression, auditory hallucinations, and mild motor delay (according to DSM-5 criteria for COS diagnosed at 6 years of age) was described by Smedemark-Margulies et al.^[81]^ Besides, 3 unrelated patients with different de novo variants in *ATP1A3* (Asp801Asn; p.Glu815Lys; p.Ala813Val) were reported by Chaumette et al.^[82]^ Two of them were diagnosed with both COS and AHC disorder, and the third was associated with COS and ASD.

### 5.4. Paroxysmal dyskinesia

Twenty-year-old monozygotic twins with a history of paroxysms of hyperkinetic movements presenting with painful abnormal postures lasting from minutes to hours up to 20 times per week were reported by Zuniga-Ramirez et al.^[83]^ The movements were triggered by weather changes, mood swings, caffeine intake, exercise, fever, infections, and speech arrest. Interictal generalized dystonic postures may affect axial muscles, upper extremities, and both legs. The same clinical features were detected in the twins but were less pronounced. In both cases, dystonic spells involved the entire body. Genetic analysis revealed a novel heterozygous *ATP1A3* p.Leu815Arg mutation. Ultimately, a family with 3 adult patients presenting with childhood paroxysmal exercise-induced dystonia in the absence of plegic attacks was reported by Roubergue et al.^[84]^

### 5.5. Cerebral palsy/spastic paraparesis

In four unrelated cases were found *ATP1A3* p.Pro775Leu mutation by Calame et al.^[85]^ All patients showed spastic dyplegia, DD, epilepsy, and episodic neurological deterioration. Among them, however, only one also showed static encephalopathy, microcephaly, and dystonia.

### 5.6. Dystonia, dysmorphism, encephalopathy, MRI abnormalities, and no Hemiplegia

Prange et al.^[86]^ described four patients with de novo heterozygous variants in *ATP1A3*. Patient 1 (c.1079C>G, p.Thr360Arg) was an 8-year-old girl who presented in the early days with episodic dystonia, complex partial seizures, facial dysmorphism, and cerebellar hypoplasia on MRI. Analogously, the patient 2 (c.420G>T, p.Gln140Lys) was an 18-year-old-boy, who was suffering since the initial days of his life from hypotonia, tremor, and facial dysmorphism and subsequently dystonia. Brain MRI revealed cerebellar hypoplasia and later cerebellar atrophy. Patient 3 (c.974G>A, Gly325Asp), a 13-year-old girl, affected by birth tremor, episodic dystonia, and facial dysmorphism. Severe cerebellar hypotrophy was also found in the brain MRI. Patient 4 (c.971A>G, p.Glu324Gly), a 14-year-old boy showed birth tremor, hypotonia, dystonia, nystagmus, facial dysmorphism, and subsequently epileptic seizures.

### 5.7. Congenital hydrocephalus and other brain abnormalities

Two germline mutations in *ATP1A3* (p.Arg19Cys and p.Arg463Cys) were reported in 2 patients by Allocco et al.^[87]^ one of which showed severe obstructive congenital hydrocephalus due to acqueductal stenosis associated with open schizencephaly, Chiari malformation 1, and dysgenesis of the corpus callosum. In a large cohort of patients, Vetro et al.^[88]^ analyzed the genetic causes of developmental and epileptic encephalopathy variably associated with cortical developmental malformations and reported 22 subjects with de novo or inherited heterozygous *ATP1A2*/*ATP1A3* mutations. Polymicrogyria was found in 10 patients (45%) with a bilateral perisylvian pattern. Early neonatal seizures with multifocal or migrating patterns were observed. A distinctive, profound phenotype, featuring polymicrogyria or progressive brain atrophy and epilepsy, was the cause of early lethality in 7 patients (32%).

## 6. Conclusions

AHC is a complex heterogeneous but distinct disorder in which hemiplegic episodes are the most relevant clinical features, with a wide variability in the frequency and intensity of episodes, and are often associated with epilepsy, dystonia, DD, and movement disorders. dystonia, and autonomic nervous system dysfunction. Most AHC cases are linked to *ATP1A3*-related disorders, including other pathologies such as RDP, CAPOS, EIEE, CROA, and RECA. New reports on disorders related to *ATP1A3* gene mutations have shown that the spectrum of *ATP1A3* disorders continues to expand with regular progression.

## Acknowledgments

We would like to express our gratitude to Prof. Lorenzo Pavone, retired the Full Professor Pediatric Clinic University of Catania, for helpful advice on various clinical issues in the article.

## Author contributions

Conceptualization: Pavone P, Parano E.

Supervision: Ruggieri, M.

Methodology: Falsaperla, R.

Writing – original draft: Pavone P, Pappalardo XG, Parano E.

Writing – review and editing: Pavone P, Pappalardo XG, Ruggieri M, Falsaperla R, Parano E.
